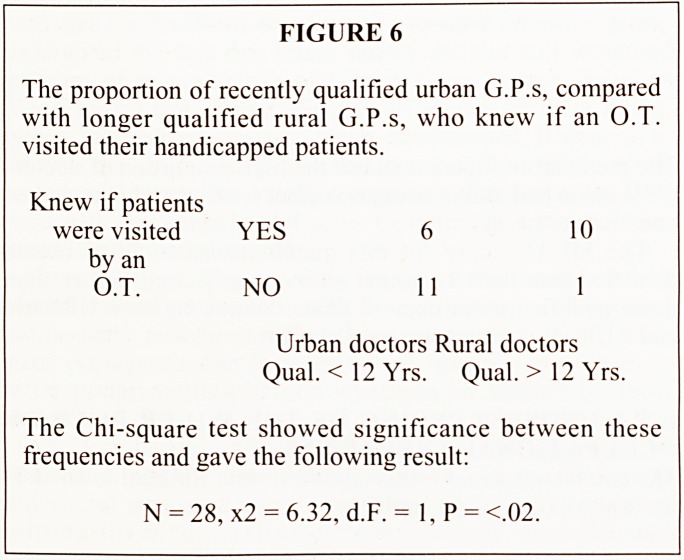# Occupational Therapy—Does the Doctor Know?

**Published:** 1992-12

**Authors:** Elizabeth Bawn

**Affiliations:** Occupational Therapist South Devon Social Services


### Occupational Therapy — Does the Doctor Know?

**Elizabeth Bawn, Dip.C.O.T., S.R.O.T.**
Occupational Therapist
South Devon Social Services

*Address for correspondence:*
Mrs. E. Bawn
4 Longfield, Lutton
Ivybridge, Devon PL21 9SN

### SYNOPSIS
With the impending implementation of Community Care and moves towards more multi-disciplinary methods of working, an O.T. working in South Devon, explored G.P.s' knowledge, about Occupational Therapy and in particular, the role of Social Services Occupational Therapists .

### INTRODUCTION
As far back as the Seebolm[1] and Tunbridge[2] reports the need had been highlighted, for family doctors to be informed about services available to them.

In 1986 a Nuffield Working Party on Communication[3] highlighted the fact that many family doctors still remained ignorant of the expertise of Occupational therapists when giving advice and assistance, upon all aspects of daily living, to those permanently or temporarily handicapped. It went on to discuss the importance of good communication between doctors and paramedical staff, recognising that the person who visits patients regularly and plans treatment programmes, has valuable information to offer.

Occupational Therapy has traditionally been a much misunderstood profession, with distant concepts of knitting and basketry now been replaced by that of providers of bath seats. As a profession they are called annually to promote a better understanding of just what can be offered by their profession.

An Occupational Therapist uses an holistic approach to assess and treat through activity, the physical, psychological and social needs of people in their chosen environment, helping them to maximise their level of function and independence in all aspects of daily life.

The 1986 Chronically Sick and Disabled Persons Act, required there to be an increase in awareness about the availability of community services and resources. 'Working for Patients' and 'Caring for People' when fully implemented will affect the way in which all medical staff organise their services. Occupational therapists will need to make their role very clear to colleagues, if best use is to be made of their expertise.

Hence this study aimed to investigate the present levels of understanding G.P.s had of the roles of their paramedical colleagues.

### METHOD
The survey was designed to measure the levels of understanding G.P.s had of both the traditional differences between Physiotherapy, Speech Therapy, and Occupational Therapy, and the wider services which might be offered by a Social Services Occupational Therapist. It also sought to discover the extent to which G.P.s referred to S.S.O.T.s, and whether they knew if S.S.O.T.s visited their severely handicapped patients.

G.P.s trained since 1978, when vocational training was introduced, had a rehabilitation component and orientation in community services included in their training. The date of this change was used as a datum for comparisons, between results. Comparisons were also made between doctors from urban and rural practices.

With no suitable questionnaire available, one to meet the objectives was designed. Initially doctors were asked when they had learnt most about O.T. Then, to measure the understanding of therapists' roles, doctors had to decide from a list of hypothetical cases, to which therapist it would be most appropriate to refer. (See Tables 1 & 2.) These questions were shown to a range of therapists whose correct results indicated that although the questions were rather subjective, they provided an accurate representation of therapists roles.

Further questions asked, if G.P.s ever referred to Social Services Occupational Therapist. (S.S.O.T.), and if G.P.s knew if any of their severely handicapped patients were visited by a S.S.O.T.

The questionnaire was sent with a covering letter of explanation, to 60 G.P.s working in South Devon.

The results were collated, mean scores calculated, and the Chi- square test used to test for significance..

### RESULTS
A response rate of 88% was recorded, with 85% asking for feedback, this suggested the questionnaire was received with interest.

Of the 53 returned questionnaires, 22 were from urban practices and 25 from rural practices; 30 had been qualified as doctors less than 12 years and 22 more than 12 years. These made suitable numbers for comparison.

**FIGURE 1**

This question was designed to establish if G.P.s understood the traditional differences between Occupational Therapy, Physiotherapy, and Speech Therapy.

**Which of the following patients would you refer to an Occupational therapist as opposed to another type of therapist (assuming availability)?**

| | Only tick boxes which refer to O.T. |
|---|---|
| (a) Man with O.A. spine needing manipulation to back. | ☐ |
| (b) Lady with M.S. needing advice on cooking from a wheelchair. | ☐ |
| (c) Man needing mobilization to knee following trauma. | ☐ |
| (d) Parents of C.P. child needing advice on property alterations. | ☐ |
| (e) Downs Syndrome child needing a special outdoor chair. | ☐ |
| (f) Dysphasic patient frustrated by inability to communicate. | ☐ |
| (g) Child of 4 yrs. with delayed speech and language development. | ☐ |
| (h) Lady needing activities to increase movement in arm after mastectomy. | ☐ |

### FIGURE 2

This question measured the wider understanding that G.P.s have of the role of the O.T.

**Which of the following patients would you expect an O.T. to be able to help?**

Only tick boxes which refer to O.T.

(a) Man with emphysema wanting advice on a stair lift. ☐

(b) Agoraphobic needing help to go shopping for family. ☐

(c) Girl with poor posture needing a gait analysis. ☐

(d) Parents needing practical help getting a disabled child into a normal school. ☐

(e) Man needing neck exercises for cervical spondylitis. ☐

(f) R.A. lady needing advice on specific bathing equipment. ☐

(g) Girl with learning difficulties who needs training in personal care ☐

(h) Frail husband caring for disabled wife needing practical and psychological support. ☐

### WHEN G.P.s LEARNT ABOUT O.T.

86% of G.P.s received most knowledge about O.T., AFTER qualifying as doctors. Of the other 14%, all had been qualified within the last 12 years. Figure 3 shows this to be a statistically significant result.

### FIGURE 3

**The proportion of G.P.s qualified MORE or LESS than 12 years. who learnt most about O.T. AFTER qualifying.**

| Years Qualified | | YES | NO |
|---|---|---|---|
| | < 12 years | 21 | 8 |
| | > 12 years | 21 | 0 |

Learnt most O.T. AFTER Qualifying.

This figure illustrates that however long qualified, the majority learnt most about O.T. after qualifying as doctors. The Chi- Square test showed significance between these frequencies and gave the following result:

N = 50, x2 = 5.00, d.F. = 1, P = <.05.

### G.P. UNDERSTANDING OF THE TRADITIONAL ROLE OF O. T. AS DISTINCT FROM OTHER THERAPIES

The correct answers for this question were:
Referral to an O.T. for (b), (d), (e), and (h).

### FIGURE 4

The pie chart in Figure 4 shows the high proportion of doctors (79%) who had all the answers correct ( + 8), or who made just one mistake ( + 6).

The MEAN score for this question was +6.00. Doctors qualified less than 12 years, on average scored higher than those qualified more than 12 years. Scores between URBAN and RURAL doctors were very similar.

### G.P.S UNDERSTANDING OF THE WIDER ROLE OF OCCUPATIONAL THERAPISTS

The correct answers for this question were: Referral to an O.T. for (a), (b), (d), (f), (g), and (h).

### FIGURE 5

The pie chart in figure 5 illustrates the broader range of scores for this question. By comparison only 38% had all the answers correct ( +8), or just one mistake (+6). The MEAN score for this question was +3.96. Doctors qualified less than 12 years, on average had higher scores than those qualified more than 12 years. Also urban doctors scored higher than rural doctors, on average.

### DOCTORS REFERRAL RATES TO SOCIAL SERVICES OCCUPATIONAL THERAPISTS

94% of doctors said they referred to this service, with the average rate being between 8 and 11 patients per doctor, per year.

### DOCTORS AWARENESS OF WHETHER THEIR SEVERELY HANDICAPPED PATIENTS WERE VISITED BY A SOCIAL SERVICES OCCUPATIONAL THERAPIST

58% of all the doctors said they were aware whether or not their patients were visited by an O.T., but 91% of the rural longer qualified doctors said they, were aware.(see Figure 6).

**FIGURE 6**

The proportion of recently qualified urban G.P.s, compared with longer qualified rural G.P.s, who knew if an O.T. visited their handicapped patients.

| | | Urban doctors Qual. < 12 Yrs. | Rural doctors Qual. > 12 Yrs. |
|---|---|---|---|
| Knew if patients were visited by an O.T. | YES | 6 | 10 |
| | NO | 11 | 1 |

The Chi-square test showed significance between these frequencies and gave the following result:

N = 28, x2 = 6.32, d.F. = 1, P = <.02.

### DISCUSSION

Bearnshaw's 'Last on the List' Kings Fund report[4], stated that with more flexible domiciliary care arrangements and improved co-ordination between agencies, practical help for people with physical disabilities could improve. This small study in South Devon suggested that there was still not a clear understanding by all G.P.s, about existing services.

From the study, those G.P. s who trained since vocational training became compulsory, had slightly better results for their understanding of the O.T. profession. However, regardless of when they trained the majority said they had learnt more about O.T. since qualifying as G.P.s.

This study found that generally doctors had a good appreciation of the traditional differences between therapists' roles, with 79% scoring well. The most common mistake being (h), the lady needing activities to increase arm movement after a mastectomy. Whereas traditionally a physiotherapist provides exercises, it would be an O.T. who would use an appropriate activity to build up muscle strength and range of movement.

The survey was conducted in an area where all Social Services O.T. staff had a similar role, which took an holistic view of clients needs. It could therefore be expected that disabled people in that area would have received a standardised service . However, the survey suggested that doctors were not all aware of the wide range of services these staff could offer their clients. Only 38% of doctors had a good score for this area of questioning. The most common mistake in this section was (b), the agoraphobic needing help to go shopping. It could reasonably be understood that maybe doctors felt a Home Help could shop for her, but in reality an O.T. could actually offer therapy to encourage the person to overcome her fears. All doctors who completed that section selected (a),and (f), and the majority also selected (h). Hence it would seem from the study, that an O.T. would be called in to advise on a stair lift provision, and equipment to overcome bathing problems, but there is less awareness of other services which could be provided. These lower scores confirmed both Busuttil's[5] and Snow's[6] research which indicated the blurring of roles and lack of awareness about certain services in community practice, and suggests that the diversity of O.T. skills were not sufficiently recognised.

Referral to Social Services O.T.s, was made by 94% of doctors, interestingly the three who didn't, all had a low score for their understanding of O.T. Referral rates varied considerably, and some doctors added comments about the shortage of O.T. staff, which led to long waiting lists for assessment, and lack of funding for equipment provision.

Despite most doctors referring to O.T., only 58% of them were aware whether or not their severely handicapped patients were visited by an O.T. It was interesting that the rural longer qualified doctors were the group to be most aware of this.

### THE WAY FORWARD

Fraser-Holland[7] highlighted the need for occupational therapists to update and educate their medical colleagues about their skills and the availability of resources. With the majority of doctors learning most about occupational therapy once qualified, an appropriate opportunity for this exchange, should be sought by therapists.

In those practices that have regular input from therapists, research has shown clearer awareness between colleagues of each others specialist skills.

With the implementation of Community Care comes the need for clear lines of communication to be established about role and availability of services, if our handicapped patients are to receive improved packages of care.

E. A. Bawn, Dip.C.O.T., S.R.O.T., 1992

### REFERENCES

1. Report of the Committee on Local Authority and Allied Personal Social Services (Seebolm Report). London: HMSO, JULY 1968. (Reprinted 1972) SBN 10 137030 X . Page No. 213.
2. Department of Health and Social Security - Welsh Office. (Central Health Services Council). Rehabilitation - Report of a Sub-Committee of the Standing Medical Advisory Committee (Tunbridge Report). London HMSO, 1972. SBN 11 3220479 5. Page 82.
3. Walton Sir John & McLachlan Gordon, Editors (1986). A collection of essays by a Nuffield Working Party on Communication: Partnenhip or Prejudice, Communication between doctors and those in the other caring professions. ISBN 0900574 61 5. Page 43.
4. Beamshaw Virginia. 'Last on the List' Community Services for People with Physical Disabilities. Research Report 3. Published by Kings Fund Institute. ISBN 187 0607 07 4. Page 46.
5. Busuttil Joseph. Community Occupational therapy and the General Practitioner. BJOT March 1985. Page 81.
6. Snow Norma. Does your G.P. really need you? Unpublished research project, 1989, Continuing Education Scheme - Exeter University. Page 17.
7. Fraser-Holland E.Naomi, The Participation of Occupational therapists in the Education of Other Professionals about Occupational therapy - A Report of a questionnaire study on behalf of the Education and Research Board of the C.O.T. BJOT September 1989. Page 338.

## Figures and Tables

**FIGURE 1 f1:**
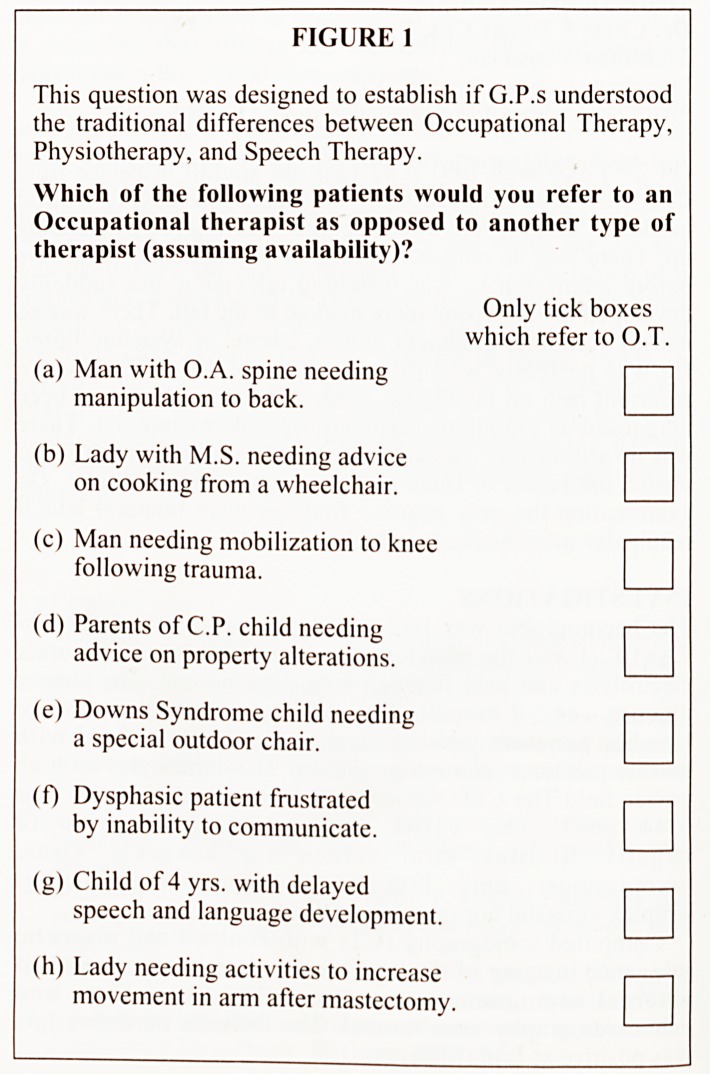


**FIGURE 2 f2:**
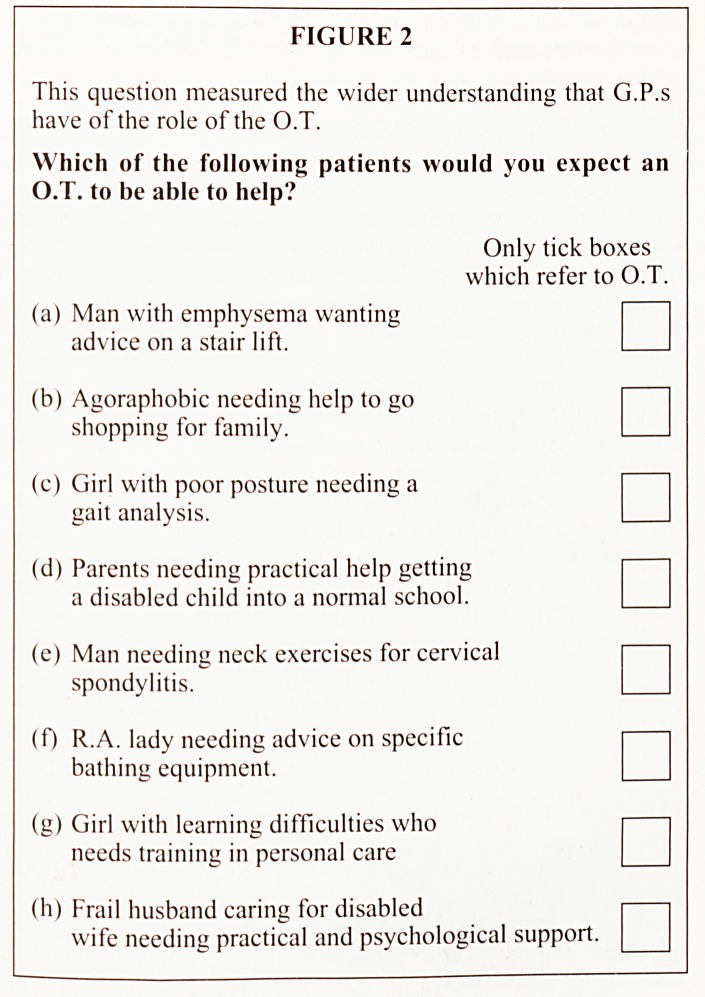


**FIGURE 3 f3:**
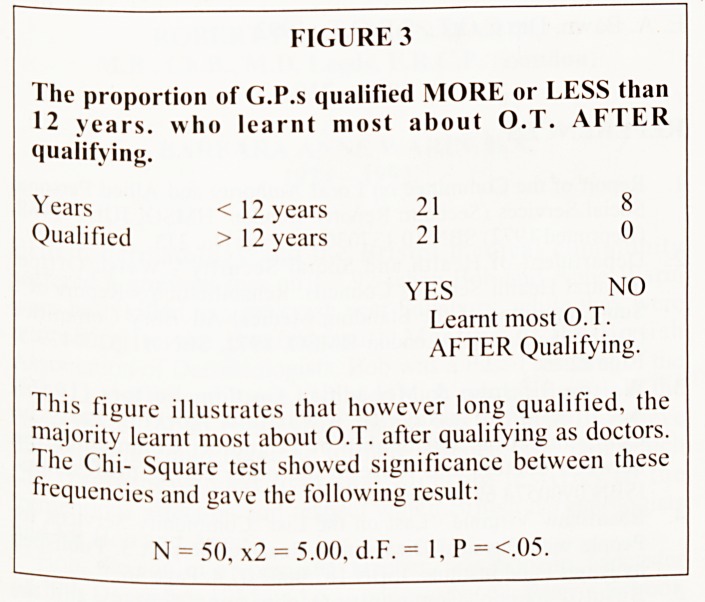


**FIGURE 4 f4:**
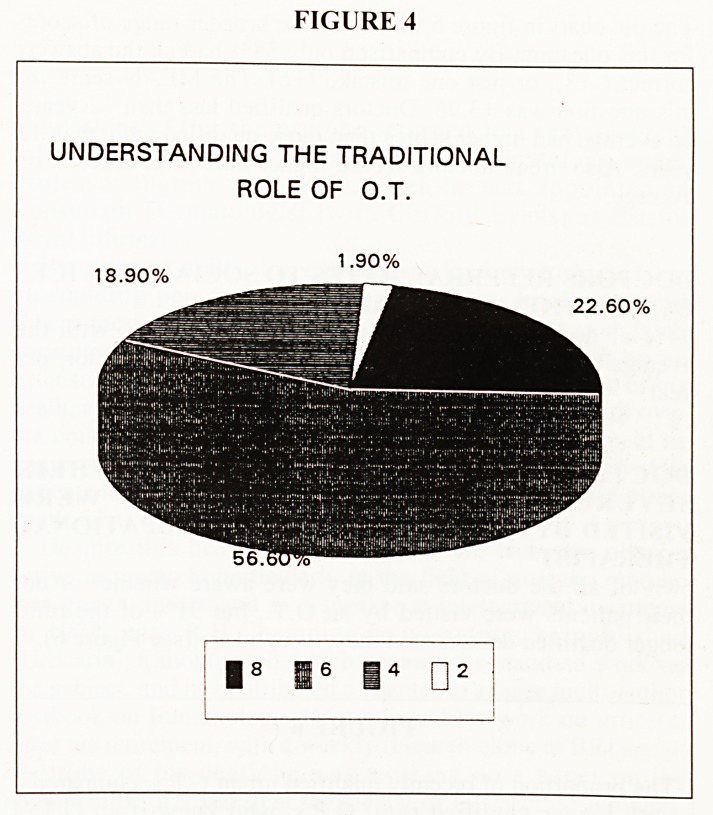


**FIGURE 5 f5:**
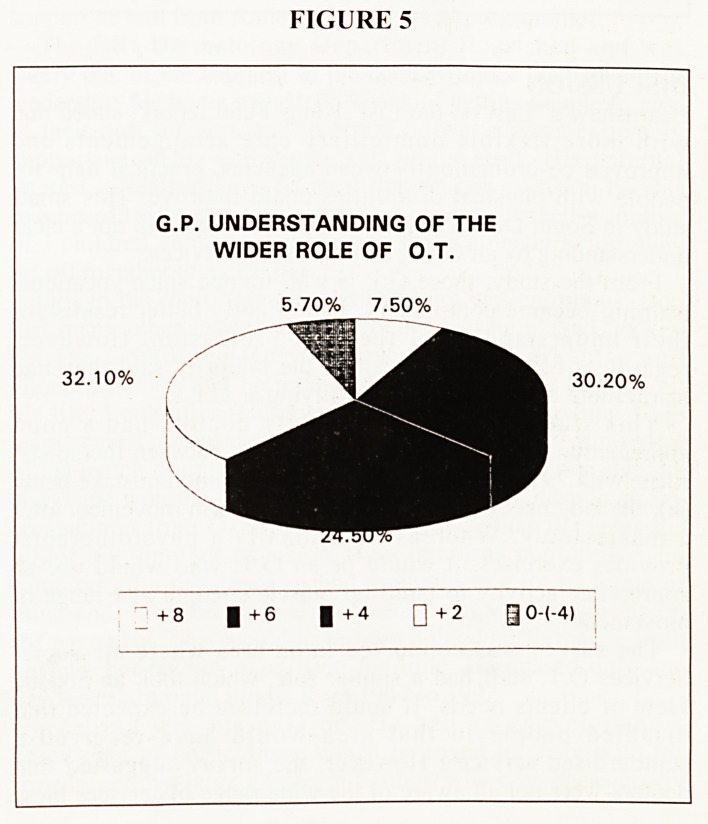


**FIGURE 6 f6:**